# Gyroid cuticular structures in butterfly wing scales: biological photonic crystals

**DOI:** 10.1098/rsif.2007.1065

**Published:** 2007-06-13

**Authors:** K Michielsen, D.G Stavenga

**Affiliations:** 1 EMBD, Vlasakker 212160 Wommelgem, Belgium; 2 Department of Neurobiophysics, University of GroningenNijenborgh 4, 9747 AG Groningen, The Netherlands

**Keywords:** structural colour, Lepidoptera, gyroid, self-assembly, cubic surfaces

## Abstract

We present a systematic study of the cuticular structure in the butterfly wing scales of some papilionids (*Parides sesostris* and *Teinopalpus imperialis*) and lycaenids (*Callophrys rubi*, *Cyanophrys remus*, *Mitoura gryneus* and *Callophrys dumetorum*). Using published scanning and transmission electron microscopy (TEM) images, analytical modelling and computer-generated TEM micrographs, we find that the three-dimensional cuticular structures can be modelled by gyroid structures with various filling fractions and lattice parameters. We give a brief discussion of the formation of cubic gyroid membranes from the smooth endoplasmic reticulum in the scale's cell, which dry and harden to leave the cuticular structure behind when the cell dies. The scales of *C. rubi* are a potentially attractive biotemplate for producing three-dimensional optical photonic crystals since for these scales the cuticle-filling fraction is nearly optimal for obtaining the largest photonic band gap in a gyroid structure.

## 1. Introduction

The colour of an animal's body is due to spectrally selective reflection of incident light (Fox & Vevers 1960). The body's reflectance spectrum is determined by the presence of absorbing pigments, and/or by submicrometre structural variations causing interference, diffraction or scattering. In the first case, the colours are referred to as pigmentary (chemical) colours and, in the second case, as structural (physical) colours. Although pigmentary colours are by far the most common, several recent studies have demonstrated that physical colours are widely employed in the animal kingdom (Srinivasarao 1999; Tayeb *et al*. 2003; Vukusic & Sambles 2003; Kinoshita & Yoshioka 2005; Welch 2005; Prum *et al*. 2006).

If the structural variations are periodic with a periodicity of the order of the wavelength of visible light, then they are often called biological photonic crystal structures. In insects, the most frequently found simple photonic crystal type is the multilayer. This one-dimensional photonic crystal consists of layers of alternating high and low variable refractive index. Multilayers are responsible for the metallic colours of the cuticle of body and elytra of many beetle species (Neville & Caveney 1969; Caveney 1971; Schultz & Rankin 1985; Parker *et al*. 1998; Kurachi *et al*. 2002). More complex multilayer structures, showing structural variations within the layers, occur in the ridges of wing scales of many butterfly species, specifically morphos (Ghiradella *et al*. 1972; Ghiradella 1991; Vukusic *et al*. 1999; Kinoshita *et al*. 2002; Wickham *et al*. 2006). Three-dimensional photonic crystals, having a three-dimensional periodic distribution of refractive indices, are found in the scales of weevils (Welch 2005) and butterflies (Morris 1975; Ghiradella & Radigan 1976; Argyros *et al*. 2002; Biró *et al*. 2003; Vukusic & Sambles 2003; Kertész *et al*. 2006; Prum *et al*. 2006).

Measured reflectance spectra of the butterfly scales with a periodic three-dimensional cuticle/air structure have been explained with qualitative and approximating approaches (Morris 1975; Biró *et al*. 2003; Kertész *et al*. 2006; Prum *et al*. 2006), but so far an adequate electromagnetic treatise has not been forwarded for any biological three-dimensional photonic crystal. One reason for the lack in progress is the uncertainty about the detailed structure of the insects' cuticle. In the case of the lycaenid species *Callophrys rubi*, first, a simple cubic (SC) network (SC ordering of air spheres in a matrix of cuticle) was assumed for the structure (Morris 1975), but later it was argued that it was more like a face-centred cubic (FCC) network (Ghiradella & Radigan 1976); a woodpile structure has been assumed in *Polyommatus* (Biró *et al*. 2003); and an FCC inverted opal structure (FCC ordering of air spheres in a matrix of cuticle) was argued to exist in the ventral wing scales of another lycaenid, *Cyanophrys remus* (Kertész *et al*. 2006). A cuticle of tetrahedral structure is assumed to exist in the papilionid species *Parides sesostris* (Vukusic & Sambles 2001) and *Teinopalpus imperialis* (Argyros *et al*. 2002).

In this paper we present an analysis of the cuticular structures of a number of butterflies, which will provide us with a solid basis for further electromagnetic calculations of the photonic crystal properties of butterfly wings. We show that several of the three-dimensional cuticular structures in the lycaenid and papilionid species, including *C. rubi*, *C. remus*, *P. sesostris* and *T. imperialis*, can be modelled by a gyroid structure, a bicontinuous triply periodic structure with a body-centred cubic (BCC) Bravais lattice symmetry. In the wings of these butterfly species, the gyroid structures have two unequal continuous subvolumes, the largest one filled with air and the smallest one filled with cuticle. We demonstrate that extensive combined transmission electron microscopy (TEM) and scanning electron microscopy (SEM) studies together with computer modelling are crucial for the characterization of the three-dimensional cuticle/air structures. Cross-checking of the information obtained by each of these three methods is inevitable to avoid erroneous structure analyses.

The paper is organized as follows. In §2, we discuss triply periodic minimal surfaces, since they are an important tool in describing cubic phases. In §3, we elaborate on cubic phases observed in lipid–water systems and biomembranes, and on the scale development in the olive hairstreak, *Mitoura gryneus*. Section 4 describes how we characterize the cubic cuticular structures in butterfly wing scales by comparing TEM micrographs from the literature with computer-generated projections modelled by level surfaces. We present a detailed analysis of the sesostris cattleheart, *P. sesostris*, and the green hairstreak, *C. rubi*, and for other members of the Papilionidae and the Lycaenidae we provide a brief summary of our results. We find that the cuticular structure in the wing scales of these butterflies can be modelled by gyroid structures with various cuticle-filling fractions and lattice parameters. In §5, we propose to use the scales of *C. rubi* as a biotemplate to produce a three-dimensional photonic crystal with a large photonic band gap (PBG) in the optical wavelength range.

## 2. Bicontinuous cubic structures

In mesoscopic self-assembled bicontinuous cubic structures, interfaces separate adjacent regions of different composition. These interfaces, often called intermaterial dividing surfaces (IMDS), are triply periodic, i.e. they are periodic along the three spatial coordinates. In bicontinuous structures, one IMDS divides the space into two distinct volumes. Each volume, or region, forms a continuous network in the system. Also, multicontinuous structures exist, but they will not be considered here. The two regions defined by the bicontinuous IMDS are not simply connected. They interpenetrate each other in a complicated way. The two regions may differ in shape or there may exist symmetry operations mapping one region onto the other. The symmetry properties of the structures are therefore described by either the crystallographic space group that does not include symmetry operations that would interchange the different regions or the space group that does include these symmetry operations.

IMDS can be approximated by constant mean curvature surfaces that can be modelled by level surfaces (Wohlgemuth *et al*. 2001). Single level surfaces, dividing space into two infinite, connected but disjunct regions, are defined by (Wohlgemuth *et al*. 2001)

(2.1) $$f(x,y,z)=\sum_{hkl}\left|F(hkl)\right|\text{cos}(hX+kY+lZ-\alpha_{hkl})=t,$$

where *X*=2*πx*/*a*, *Y*=2*πy*/*a*, *Z*=2*πz*/*a*; (*x*, *y*, *z*) are the positions in the crystal structure; *a* denotes the length of the cubic unit cell; |*F*(*hkl*)| denotes the structure factor amplitude, reflecting the symmetry of the structure, and *α*_*hkl*_ denotes the phase angle, where (*h k l*) are the positions in the reciprocal lattice; and the parameter *t* determines the volume fraction of the two regions. For *t*=0, equation (2.1) defines the nodal surfaces (von Schnering & Nesper 1991). Nodal surfaces are used as approximations to the triply periodic minimal surfaces, surfaces for which the mean curvature is zero at every point. The term ‘minimal surface’ originates from experiments conducted by the nineteenth century physicist Plateau. He immersed various metal frames, which were not necessarily planar, in soap solutions. The soap films spanned in the frames are such that the surface free energy is minimal, that is, such that the area of the films is minimal (Plateau 1873).

There are three fundamental cubic (minimal) surfaces: the primitive or P-surface; the diamond or D-surface; and the gyroid or G-surface, with space groups $Im\bar{3}m$, $Pn\bar{3}m$ and $Ia\bar{3}d$, respectively. The level (nodal) surfaces modelling these cubic (minimal) surfaces are given by (von Schnering & Nesper 1991; Wohlgemuth *et al*. 2001)

(2.2) P:cosX+cosY+cosZ=t,

(2.3) D:cosZsin(X+Y)+sinZcos(X−Y)=t,

(2.4) G:sinXcosY+sinYcosZ+cosXsinZ=t.

**Figure 1:**
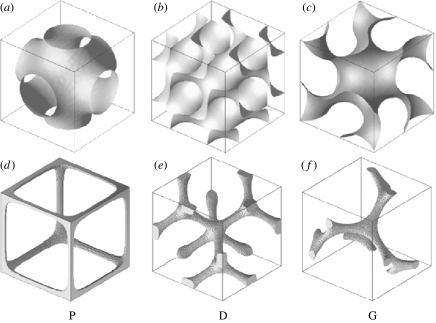
(*a–c*) Unit cells of the P, D and G nodal surfaces constructed from equations (2.2)–(2.4), respectively, with *t*=0. These surfaces divide the cubic structure into two regions forming two continuous interpenetrating networks in the system. (*d–f*) Skeletal graphs for one of the two distinct networks formed by the P, D and G nodal surfaces. The skeletal graphs show the connectivity of the channels in the network. In the P-, D- and G-structures, these channels are six-, four- and threefold connected, respectively.

These surfaces divide the cubic structure into two regions, forming two distinct interpenetrating networks. In the P-structure, the channels in a particular network are sixfold connected, in the D-structure they are tetrahedrally connected and in the gyroid the channels join as triads. The gyroid is chiral, the two channels have different handedness, which are related by an inversion. The unit cells of the P, D and G nodal surfaces (*t*=0) together with the skeletal graphs, showing the connectivity of the channels in the network, are depicted in figure 1.

The level surfaces only subdivide space into two continuous subvolumes for a certain interval of *t*-values (Lambert *et al*. 1996). At the boundaries of this interval, the surfaces ‘pinch-off’, i.e. they become disconnected and reduce to a lattice of closed-packed units with a given symmetry (Anderson *et al*. 1990; Lambert *et al*. 1996).

There also exist more complex cubic surfaces (von Schnering & Nesper 1991; Wohlgemuth *et al*. 2001), but so far there is no convincing evidence for their existence in mesoscopic self-assembled bicontinuous structures.

## 3. Self-assembly, membranes and cell biology

Before discussing the processes that might lead to the formation of cubic lattice structures in butterfly wings, we first elaborate on the cubic phases observed in lipid–water systems and biomembranes.

### 3.1 Lipid–water systems

Lipid bilayers are ubiquitous in biological membranes, including plasma membranes. If the constituent monolayers in the lipid bilayer are made up of identical molecules, based on geometrical constraints, the mid-surface of the bilayer is expected to be a quasi-homogeneous minimal surface (Hyde *et al*. 1997). The simplest examples of these minimal surfaces are the triply periodic minimal surfaces having cubic symmetry (Hyde *et al*. 1997). Among them are the P-surface, the D-surface and the gyroid (or G-surface), which can be formed with the least bending energy cost, that is, they minimize the curvature energy (Hyde *et al*. 1997). Hence, as a result of self-assembly, a structure is made in which the lipid bilayer separates two water channels. If the water channels on both sides of the bilayer are different, or the lipid bilayer contains constituent monolayers of different curvature, an asymmetric bicontinuous cubic phase having constant but non-zero mean curvature results. Although these triply periodic structures can be difficult to express in rigorous mathematical terms, they can be well approximated by level (nodal) surfaces (§2).

Geometrical constraints are not sufficient to predict the occurrence of bicontinuous cubic phases in lipid–water systems. Also, the temperature and the concentration of the mixture play a role. In lipid–water phase diagrams (e.g. Larsson 1989), in general, three types of phases, having respectively one-, two- and three-dimensional periodicity, can be identified, namely the lamellar phase in which water layers are alternating with lipid bilayers, the hexagonal phase in which infinite water cylinders are arranged in a hexagonal array and are separated by lipid bilayers, and the cubic phase consisting of curved infinite lipid bilayers. Upon sufficient heating or increasing the water content, a transition occurs from the lamellar to the cubic phase and finally to the hexagonal phase (Larsson 1989).

Bicontinuous cubic phases in pure lipid–water systems were first detected by Luzzati and co-workers (Luzzati & Spegt 1967; Luzzati *et al*. 1968). Longley & McIntosh (1983) have shown that in lipid–water systems, both gyroid- and D-surface structures can be observed. At lower water contents, the G-surface structure is formed and at higher water contents the D-surface structure is formed. The same type of cubic phase has been observed in ternary lipid–protein–water systems (Ericsson *et al*. 1983). In these systems, the protein molecules are located in the water channels. Later, it was shown that with further increasing water content the P-surface structure also exists in these ternary lipid–protein–water systems (Buchheim & Larsson 1987).

More detailed information about cubic phases in lipid-containing systems and their possible biological relevance can be found in, for example, Lindblom & Rilfors (1988), Mariani *et al*. (1988), Larsson (1989), Fontell (1990), Luzzati *et al*. (1993) and Hyde *et al*. (1997).

### 3.2 Cubic membranes

Cytomembranes contain a variety of lipids and proteins. Most simply, we could think of the lipids being in a planar bilayer state. Changing physico-chemical conditions (temperature and/or changes in local solution environment) may however lead to a curved cubic phase. In contrast to pure lipid–water mesostructures, cytomembranes have an inherent bilayer asymmetry. Hence, the resulting cubic membranes will have a constant but non-zero mean curvature.

Cubic membranes have a widespread occurrence throughout the animal and plant kingdoms (e.g. Hyde *et al*. 1997). Cubic membranes can evolve virtually from any cytomembrane, i.e. the plasma membrane, the rough and the smooth endoplasmic reticulum including the nuclear envelope, the mitochondria, lysosomes and the Golgi complex (Hyde *et al*. 1997). However, it seems that cubic membranes are most frequently formed in the endoplasmic reticulum (Hyde *et al*. 1997; Almsherqi *et al*. 2006).

Cubic membranes are in general formed from a structural template, the precursor to the cubic membrane, such as the invaginations of the plasma membrane (Hyde *et al*. 1997). The specific mechanisms leading to membrane folding into a cubic membrane are not known (Hyde *et al*. 1997; Almsherqi *et al*. 2006).

### 3.3 Scale development in the olive hairstreak, *M. gryneus*

The green scales of *M. gryneus* are known to have a three-dimensional lattice structure of chitin (Ghiradella 1989, 1991; Prum *et al*. 2006). In order to understand how a cell can produce such lattice structures, Ghiradella (1989) made a developmental study of the scales of the olive hairstreak, *M. gryneus*. Each scale originates from an individual cell in its wing epithelium. First, the ridges and crossribs of the scale are formed. Later, membrane–cuticle units with regular diameter are formed inside the cytoplasm. The membranes of these units appear to be invaginations of the plasma membrane (Ghiradella 1989). Thus, the spaces enclosed by these membranes are continuous with the extracellular space and the cuticle formation is extracellular. The smooth endoplasmic reticulum appears to act as a template around which the membrane–cuticle units and a bit of cytosol are wrapped (Ghiradella 1989). When the cell dies back into the epithelium of the wing, the newly formed scale dries and hardens to leave the cuticular structure behind.

Chitin, a biopolymer, is a major component of the insect's cuticle. It functions as light but mechanically strong scaffold material and is always associated with cuticle proteins that mainly determine the mechanical properties of the cuticle (Merzendorfer & Zimoch 2003). The specific mechanism by which chitin is produced is still unknown, but various models have been presented in which nascent chitin has to be transported across the plasma membrane (Merzendorfer & Zimoch 2003).

The lattice development in *M. gryneus* is consistent with the cubic membrane formation in other biological systems, which are induced by lipid and protein alternations and/or other physico-chemical changes. However, the specific mechanisms leading to the lattice formation are only poorly understood. More detailed studies of membrane transitions in living organisms are required to obtain a better understanding of the cubic membrane formation.

## 4. Cuticular structure characterization in butterfly wing scales

The cuticular structure in insects is generally investigated by means of SEM and TEM. SEM micrographs of cut surfaces give an impression of the three-dimensional character of the structure, while TEM micrographs of thin sections provide a two-dimensional view. TEM sections are usually random cuts through the structure and have a thickness of 60–100 nm. Such random cuts through a periodic cubic structure can lead to complex patterns in the TEM micrograph. The patterns do not only depend on the orientation of the section, the thickness of the section and the viewing angle, but they can also look very similar for cubic structures belonging to different space groups (Hyde *et al*. 1997). Moreover, the cubic cuticular structures can also be polymorphic and artefactually distorted. Structure determination from the observation of a single section is therefore extremely difficult. However, as we demonstrate in the following subsections, in most cases a satisfactory characterization of cubic cuticular structures can be obtained if SEM and TEM studies are combined, if SEM micrographs are made for tilted and rotated samples, if the TEM micrographs are made for various sections through the structure, and if the TEM micrographs are compared with computer-generated projections modelled by equations (2.2)–(2.4) and (4.1).

An alternative approach to study the three-dimensional cuticular structure is to make use of electron tomography combined with computer-based modelling and visualization (Argyros *et al*. 2002). In §4 we briefly compare the results obtained with both methods for the characterization of the three-dimensional cuticular structure in the wing scales of the Kaiser-i-Hind butterfly, *T. imperialis*.

### 4.1 Method

We have investigated the wing scales of various butterflies by using published SEM and TEM micrographs (Ghiradella & Radigan 1976; Ghiradella 1984, 1985, 1989, 1991, 1994; Vukusic & Sambles 2001, 2003; Argyros *et al*. 2002; Kertész *et al*. 2006; Prum *et al*. 2006).

A basic assumption of our approach is that we model the cubic air–cuticle structures of the scales by structures based on the level surfaces given by equations (2.2)–(2.4). Hence, we introduce the function

(4.1) $$g(x,y,z)=\{\begin{array}{ll} 1, & \text{if}\;f(x,y,z)<t\\ 0, & \text{if}\;f(x,y,z)\geq t \end{array},$$

where *g*(*x*, *y*, *z*)=1 denotes a filling with cuticle and *g*(*x*, *y*, *z*)=0 denotes a filling with air. Varying the parameter *t* changes the composition of the material, i.e. the amount of cuticle. The computer-generated cubic structures consist of voxels that are either coloured black (cuticle) or white (air). The black voxels have value 1 and the white voxels have value 0.

The cubic structure contains several unit cells of the P-, D- and G-structures. The P-, D- and G-structures have an SC, an FCC and a BCC Bravais lattice symmetry. We have cut sections of a given thickness *d* and with a prescribed orientation from this three-dimensional model structure, just as microtome cuts are made for TEM studies.

In order to compute the two-dimensional projections of these sections, for comparison with the TEM micrographs, we have to consider the interaction of the specimen with the electron beam. TEM micrographs of butterfly wing scales have little to no greyscale contrast and can be considered to be black-and-white images. This is because the chitin structures act as strong electron-absorbing objects so that the contrast of the image is not sensitive to focus (Argyros *et al*. 2002). Therefore, to describe the interaction of the specimen with the electron beam, we can treat the cuticle as completely opaque. This makes modelling and interpretation more straightforward than in the case of grey-scale TEM images (Anderson *et al*. 1992; Deng & Mieczkowski 1998). Hence, we compute the two-dimensional projections by summing up the values of the voxels along a line parallel to the viewing direction. If the sum differs from zero, we give the corresponding pixel in the two-dimensional projection the value 1 (black) and otherwise 0 (white).

As a final step, we have compared the TEM micrographs with computer-generated TEM images for various values of *t* and *d* and for various orientations.

### 4.2 The sesostris cattleheart, *P. sesostris*

**Figure 2:**
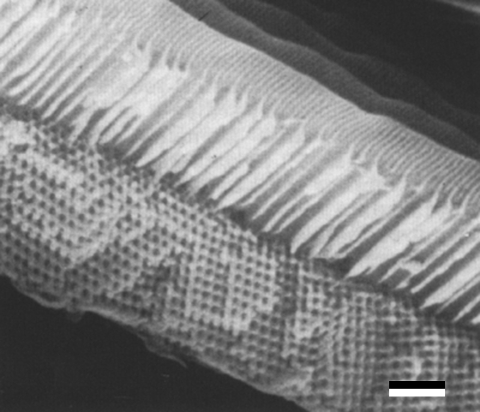
SEM micrograph showing a longitudinal view of a fractured scale of *P. sesostris*. (Reproduced with permission from Ghiradella (1994).) Scale bar, 1 μm.

As a first example, we consider the papilionid *P. sesostris*, which exhibits green spots on its ventral wings. In these regions, SEM micrographs display a regular three-dimensional lattice structure (Ghiradella 1985, 1994, 1999; Vukusic & Sambles 2001, 2003). Figure 2 shows that the rather large circular air holes in the cuticular network appear to be positioned on square (bottom right of micrograph) or triangular (centre of micrograph) lattices.

**Figure 3:**
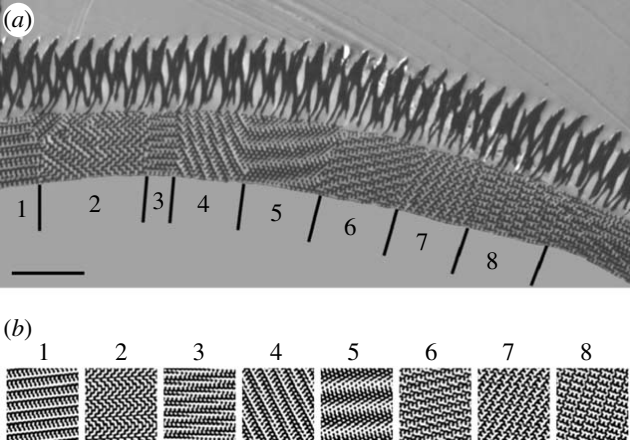
(*a*) TEM micrograph showing a cross section through the scale of *P. sesostris*. (Reproduced with permission from Vukusic & Sambles (2003).) The numbers indicate various domains in the cuticle crystal. The vertical lines indicate the position of the grain boundaries. Scale bar, 2.5 μm. (*b*) Computer-simulated projections for a G-structure with *t*=−0.3 (equations (2.4) and (4.1)). The projections are generated from sections with a thickness of 0.15*a* (*a* denotes the length of the cubic unit cell) along the directions: 1, [1 2 3]; 2, [1 1 11]; 3, $\left[\bar{4}\;8\;\bar{11}\right]$; 4, [4 9 12]; 5, $\left[\bar{1}\;9\;\bar{12}\right]$; 6, [2 4 9]; 7, $\left[7\;\bar{10}\;\bar{12}\right]$; 8, [7 7 10]. The chitin (cuticle) structures appear black in both the micrograph and the simulation.

We can gain more information about the lattice structure from TEM micrographs (Ghiradella 1985, 1991; Vukusic & Sambles 2001, 2003; Prum *et al*. 2006). Figure 3*a* illustrates that the crystal lattice structure consists of various domains with an average size of approximately 3.7 μm. In figure 3*a* we indicate eight domains that allow structural characterization.

**Figure 4:**
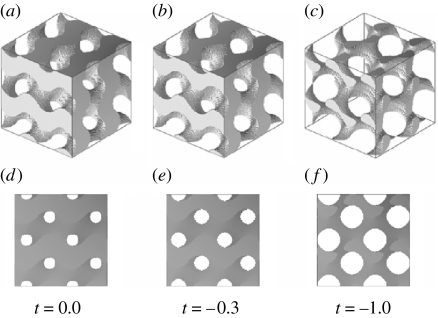
(*a–c*) Gyroid structure (eight unit cells) constructed from equations (2.4) and (4.1). For *t*=0.0, −0.3 and −1.0, the cuticle volume fractions are 0.50, 0.40 and 0.17, respectively. (*d–f*) Projections of the gyroid structures along the direction [0 0 1]. For *t*=0.0, −0.3 and −1.0, the circular holes have a diameter of approximately *a*/4, *a*/3 and *a*/2, respectively.

Simulating sections of zero thickness of P-, D- and G-structures for various values of *t* immediately suggests that the black-and-white patterns observed in the various domains are best matched by projections of a gyroid structure. Modelling gyroid structures for various values of *t*, and viewing through them in the [0 0 1] direction, shows a square lattice of circular air holes with lattice parameter $a/\sqrt{2}$. The smaller *t* becomes, the larger the air holes become and, hence, the less cuticle is present in the structure. For *t*=−0.3, the air holes have a diameter of approximately *a*/3 and the cuticle volume is 0.40 (figure 4). The square lattice of holes resembles the square lattice of holes observed in the SEM images rather well (figure 2). Viewing through the gyroid structure with *t*=−0.3 along the [1 1 1] direction shows a triangular lattice of holes that looks similar to the triangular lattice seen in the SEM image in figure 2. Choosing *t*=−0.3 and varying the thickness *d* of the sections, we have computed many projections for various viewing angles. For *d*=0.15*a* (see figure 3*b*), we achieve excellent matches with the black-and-white patterns of the eight domains of figure 3*a*. Using the same parameters (*t*=−0.3 and *d*=0.15*a*), we can also reproduce the patterns observed in the TEM micrographs reported by Ghiradella (1985, 1991) and Prum *et al*. (2006). However, the micrographs presented by Prum *et al*. (2006) are so small that various projections match.

Among the possible cubic structures that we have investigated for the description of the lattice in the scales of *P. sesostris*, the gyroid structure with *t*=−0.3 (chitin volume fraction of 0.40) alone accounts for all structural features that are available to us. Based on the comparison between the real and simulated TEM micrographs, we estimate that the lattice parameter of the gyroid network is approximately *a*=260±63 nm. Our conclusion that *P. sesostris* scales have a gyroid structure deviates from Vukusic & Sambles (2001), who concluded that a tetrahedral structure, namely that of a diamond lattice with approximately 40% occupancy, describes the lattice of cuticle, but the achieved matches were reported to be good, although not exact (Vukusic & Sambles 2001).

### 4.3 The green hairstreak, *C. rubi*

The second example we consider is the lycaenid *C. rubi*, which displays a uniform green colour over the whole of the underside of the wing. Morris (1975) was the first to examine the structure responsible for this colour by studying whole mounts of the wing scales applying light microscopy and TEM. He concluded that single wing scales are composed of a mosaic of irregular polygonal domains, with grain diameter 5.4 μm, and that a SC network with an average thickness of four lattice units could account for the variety of patterns observed inside the domains. The lattice parameter of the cubic network was estimated to be *a*=257±25 nm (Morris 1975). Based on the average value of 1.065 for the refractive index (Morris 1975), we estimate the volume fraction for the cuticle to be 0.13. The sketch of the proposed SC network (Morris 1975) can be modelled by the level surface equation (2.2), with −1.4<*t*<−1.0. For these values of *t*, the volume fraction of the cuticular network varies between 0.16 and 0.26.

**Figure 5:**
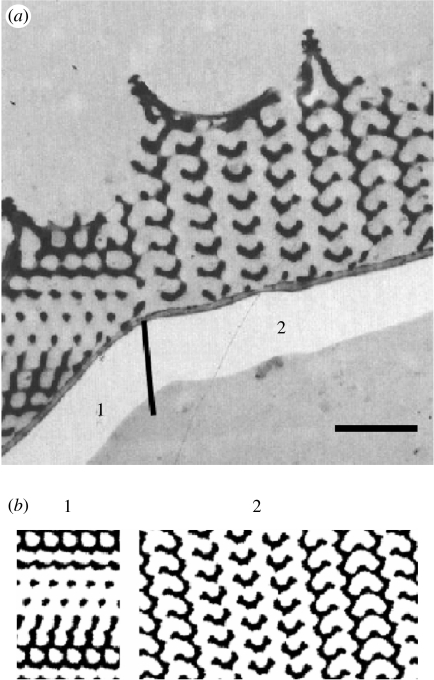
(*a*) TEM micrograph showing a transverse section through a green scale of *C. rubi*. (Reproduced with permission from Ghiradella & Radigan (1976).) The numbers indicate various domains in the cuticle crystal. The vertical line indicates the position of the grain boundary. Scale bar, 1 μm. (*b*) Computer-simulated projections for a G-structure with *t*=−1.0 (equations (2.4) and (4.1)). The projections are generated from sections with a thickness of 0.2*a* (*a* denotes the length of the cubic unit cell) along the directions: 1, $\left[\bar{1}\;6\;\bar{7}\right]$; 2, [0 3 10]. The chitin (cuticle) structures appear black in both the micrograph and the simulation.

**Figure 6:**
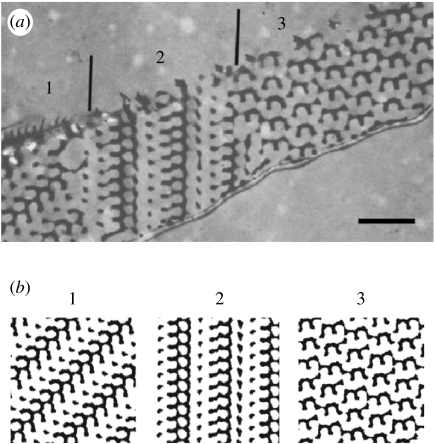
(*a*) TEM micrograph showing a longitudinal section through a green scale of *C. rubi*. (Reproduced with permission from Ghiradella & Radigan (1976).) The numbers indicate various domains in the cuticle crystal. The vertical lines indicate the position of the grain boundaries. Scale bar, 1 μm. (*b*) Computer-simulated projections for a G-structure with *t*=−1.0 (equations (2.4) and (4.1)). The projections are generated from sections with a thickness of 0.2*a* (*a* denotes the length of the cubic unit cell) along the directions: 1, [0 3 5]; 2, [2 7 9]; 3, $\left[\bar{3}\;4\;\bar{12}\right]$. The chitin (cuticle) structures appear black in both the micrograph and the simulation.

More elaborate TEM studies by Ghiradella & Radigan (1976), in which, apart from whole mounts, longitudinal and transverse sections were also examined, indicated that the internal cuticular lattice was FCC and not SC. Figures 5*a* and 6*a* with transverse and longitudinal sections show (parts of) a few crystal domains.

Based on our analysis for *P. sesostris*, we assume that the cuticular structure responsible for the green colour of *C. rubi* is also a gyroid structure, having a BCC lattice symmetry. We use this assumption to simulate the patterns observed in TEM micrographs of *C. rubi* (Ghiradella & Radigan 1976; Ghiradella 1984).

**Figure 7:**
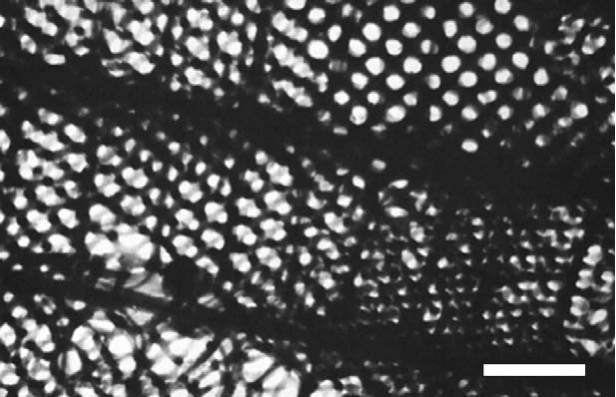
TEM micrograph of whole mount of *C. rubi* scale. (Reproduced with permission from Ghiradella & Radigan (1976).) Scale bar, 1 μm.

TEM micrographs of whole mounts (Morris 1975; Ghiradella & Radigan 1976) provide to some extent the same information as SEM micrographs since they show frontal views. In these micrographs, square lattices of air holes are rather common, but also triangular lattices can be observed (figure 7). The square lattice of holes observed in the whole-mount TEM image of Ghiradella & Radigan (1976) (figure 7) can be modelled by the frontal view (view along the [0 0 1] direction) of a gyroid structure with *t*=−1.0. In that case, the circular air holes have a diameter of approximately *a*/2 and the cuticle volume is 0.17 (figure 4). From this TEM image, the lattice unit can then be estimated, and we find *a*=363±45 nm.

Choosing *t*=−1.0 and varying the thickness *d* of the sections, we have computed several projections for various viewing angles. For *d*=*a*, many of these projections show (nearly) square lattices with (nearly) circular air holes. These projections, which can be compared with whole-mount TEM micrographs, explain why Morris (1975) misinterpreted the structure as SC with a lattice parameter of 257 nm (which is exactly $a/\sqrt{2}$). Figures 5*b* and 6*b* demonstrate that for *d*=0.2*a*, we can reproduce the black-and-white patterns and thus characterize the domains very well. Using the parameters *t*=−1.0 and *d*=0.2*a*, we can also fully reproduce the patterns observed in the TEM micrograph of a transversely sectioned wing scale of *C. rubi* in Ghiradella (1984). A comparison of the simulated and real TEM micrographs of transverse and longitudinal sections yields that the lattice parameter of the gyroid network is approximately 300 nm, which is slightly below our estimate based on the analysis of the whole-mount TEM micrograph.

In summary, we find that the cuticular lattice structure in the scales of *C. rubi* approximates the gyroid structure with *t*=−1.0 (chitin volume fraction of 0.17).

### 4.4 Other butterflies and insects

We have investigated whether our conclusion that *P. sesostris* and *C. rubi* have a gyroid cuticular structure also applies to other Papilionidae and Lycaenidae.

For the papilionid *T. imperialis*, we find that the three TEM micrographs presented by Argyros *et al*. (2002) can be generated from sections of a gyroid structure with *t*=−0.3 and thickness 0.2*a* (chitin volume fraction of 0.31), but also from sections of a gyroid structure with *t*=−0.6 and thickness 0.3*a* (chitin volume fraction of 0.31; results not shown). Such gyroids, chiral cubic structures having threefold connectivity, are completely different from the chiral tetrahedral structure found by Argyros *et al*. (2002) by means of electron tomography and computer modelling and visualization. This again shows that making a unique structure characterization of these cuticular structures remains a difficult task. Unfortunately, we were unable to find enough TEM micrographs in the literature to allow a more detailed analysis for *T. imperialis*.

The same holds for lycaenids other than *C. rubi*. The cuticular structure in the ventral wing scales of *C. remus* has been identified as an FCC inverted opal structure (Kertész *et al*. 2006). However, the underlying TEM micrograph (fig. 7 of Kertész *et al*. 2006) can be well modelled by a gyroid with *t*=−0.5 (chitin volume fraction of 0.34; results not shown). Also, the cuticular structures of *M. gryneus* (Ghiradella 1989; Prum *et al*. 2006) can be modelled by a gyroid with *t*=−1.0 and those of *Callophrys dumetorum* (Prum *et al*. 2006) by a gyroid with *t*=−0.6 or −0.3 (results not shown).

Ghiradella (1984) suggested that sections of the green scales of the weevil *Polydrusus sericeus* show the same type of lattice as that of *C. rubi*, which she assumed to be an FCC lattice of air spheres embedded in a matrix of cuticle. We however conclude, based on our analysis of the single TEM micrograph showing a frontal section (Ghiradella 1984), that the lattice structure is definitely not a gyroid structure (BCC), the structure we found for *C. rubi*, but rather a D-surface (diamond) structure (FCC).

## 5. Biological photonic crystals

In §4 we have shown that the cuticular microstructure in the wing scales of various Papilionidae and Lycaenidae species can be modelled by a gyroid structure. In the various species that we have investigated, the cuticle volume fraction varies between 0.17 and 0.40. It appears to be difficult to determine the lattice parameter from the published SEM and TEM micrographs, but we estimate that it varies between 165 and 360 nm.

It is well known that the gyroid structure has potential as a photonic crystal, a periodic dielectric composite structure with a periodicity of the order of the wavelength of electromagnetic waves, that forbids propagation for a certain frequency range, called the PBG (Martín-Moreno *et al*. 1999; Babin *et al*. 2002; Maldovan *et al*. 2002; Michielsen & Kole 2003). In general, the characteristics of PBGs in periodic dielectric structures depend on the dielectric contrast between the composites, the symmetry and topology of the structure and the filling factor, i.e. the ratio between the volume occupied by each dielectric with respect to the total volume of the composite. If for the frequencies in the PBG the propagation of electromagnetic waves is forbidden in any direction and for any polarization, the PBG is called complete, and otherwise it is called partial.

Computer simulations have shown that for relatively large dielectric contrasts (*n*/*n*′≈3.5, where *n* and *n*′ denote the refractive indices of the two composites), the gyroid structure has a complete PBG for filling factors between approximately 0.04 and 0.55 (Martín-Moreno *et al*. 1999; Babin *et al*. 2002; Maldovan *et al*. 2002; Michielsen & Kole 2003). The higher the filling factor, the lower is the midgap frequency. The largest gap is observed for a filling factor of approximately 0.20, with a midgap frequency *ωa*/2*πc*≈0.5. Hence, for these high dielectric contrasts, PBGs in the optical regime are expected for lattice parameters *a*≈200–350 nm. The large complete PBG closes for a refractive index contrast *n*/*n*′≤2.5 (Martín-Moreno *et al*. 1999; Babin *et al*. 2002; Maldovan *et al*. 2002).

The cuticle/air structures of butterfly wing scales have a refractive index contrast *n*/*n*′=(1.55±0.05)+*i*(0.06±0.05) (Vukusic *et al*. 1999). Hence, given that for various papilionids and lycaenids these structures can be modelled by a gyroid structure with a cuticle volume fraction varying between 0.17 and 0.40, we conclude that these structures are biological photonic crystals without a complete PBG. However, partial band gaps may exist for light propagation in some directions or for some polarizations. Owing to the absence of complete PBGs, it might be expected that the reflected light from the gyroid cuticular structures is strongly angle dependent. However, this effect can be masked by the fact that one scale is constructed from several domains with different orientations (§4), causing a uniform colour.

Many applications of photonic crystals are in the visible (400–700 nm) or near-infrared (500–1300 nm) wavelength range. Finding three-dimensional photonic structures with a large absolute PBG in the visual or near-infrared frequency range, which is suitable for large-scale production, is therefore highly desirable. Owing to the submicrometre resolution required in the production technology, manufacturing these photonic crystals is a great challenge. One possible way to construct three-dimensional photonic crystals in the optical wavelength range could be by using the gyroid cuticular structure in the wing scales of some Papilionidae and Lycaenidae as a template. Although it is a challenge to replicate the gyroid structure of this biotemplate, it might also be a useful tool to produce three-dimensional photonic crystals. If possible, coating of the biotemplate with a material having a higher refractive index than the one of chitin could even produce a three-dimensional photonic crystal with a large complete PBG. For this purpose, the scales of *C. rubi* could serve as a proper biotemplate, since the cuticle filling fraction is nearly optimal for obtaining the largest PBG. Very recently, a controlled replication by means of a low-temperature atomic layer deposition process was performed to produce aluminium oxide replicas of wing scales from a *Morpho peleides* butterfly (Huang *et al*. 2006). The blue colour of the wings of *M. peleides* results from a two-dimensional photonic crystal slab consisting of arrays of rectangles formed by lamellae and microribs (Huang *et al*. 2006). Although the photonic structure in *M. peleides* is simpler than the one in *C. rubi*, the same or a similar technique (Seshadri & Meldrum 2000; Cook *et al*. 2003) could be applied to produce a replica of the wing scales of *C. rubi*.
